# Silver Nanoparticles in the Water Environment in Malaysia: Inspection, characterization, removal, modeling, and future perspective

**DOI:** 10.1038/s41598-018-19375-1

**Published:** 2018-01-17

**Authors:** Achmad Syafiuddin, Salmiati Salmiati, Tony Hadibarata, Ahmad Beng Hong Kueh, Mohd Razman Salim, Muhammad Abbas Ahmad Zaini

**Affiliations:** 1 Department of Environmental Engineering, Faculty of Civil Engineering, Universiti Teknologi Malaysia, 81310 UTM Johor Bahru, Johor Malaysia; 2 Centre for Environmental Sustainability and Water Security (IPASA), Research Institute for Sustainable Environment, Faculty of Civil Engineering, Universiti Teknologi Malaysia, 81310 UTM Johor Bahru, Johor Malaysia; 3 Department of Environmental Engineering, Faculty of Engineering and Science, Curtin University, 98009 Miri Sarawak, Malaysia; 4 Construction Research Centre (CRC), Institute for Smart Infrastructure and Innovative Construction (ISIIC), Faculty of Civil Engineering, Universiti Teknologi Malaysia, 81310 UTM Johor Bahru, Johor Malaysia; 5 Centre of Lipids Engineering and Applied Research (CLEAR), Universiti Teknologi Malaysia, 81310 UTM Johor Bahru, Johor Malaysia

## Abstract

The current status of silver nanoparticles (AgNPs) in the water environment in Malaysia was examined and reported. For inspection, two rivers and two sewage treatment plants (STPs) were selected. Two activated carbons derived from oil palm (ACfOPS) and coconut (ACfCS) shells were proposed as the adsorbent to remove AgNPs. It was found that the concentrations of AgNPs in the rivers and STPs are in the ranges of 0.13 to 10.16 mg L^−1^ and 0.13 to 20.02 mg L^−1^, respectively, with the highest concentration measured in July. ACfOPS and ACfCS removed up to 99.6 and 99.9% of AgNPs, respectively, from the water. The interaction mechanism between AgNPs and the activated carbon surface employed in this work was mainly the electrostatic force interaction via binding Ag^+^ with O^−^ presented in the activated carbon to form AgO. Fifteen kinetic models were compared statistically to describe the removal of AgNPs. It was found that the experimental adsorption data can be best described using the mixed 1,2-order model. Therefore, this model has the potential to be a candidate for a general model to describe AgNPs adsorption using numerous materials, its validation of which has been confirmed with other material data from previous works.

## Introduction

In the past several years, the development of nanotechnology has marched progressively in numerous fields of application such as electronics, biological sensors, and water treatments. One of the recent advances is the development of silver nanoparticles (AgNPs) for various consumer products such as detergents, textiles, cosmetics, sprays, paints, and metal products due to their remarkable physical, chemical, and biological features^1–7^. It was estimated that the worldwide production of AgNPs was about 500 tons per year^8^. This implies that AgNPs are widely utilized on a global scale. Of central concern to the industry stakeholders, policymakers, communities, as well as researchers, however, is their disposal into the water environment such as rivers. This is because there is an accumulated evidence demonstrating that AgNPs are toxic to the aquatic animals such as bacteria^7,9^, algae^10^, and plankton^11^. They also possess the capability to alter their surrounding beneficial microbial communities^12^. Since the vast majority of the sources for drinking and household uses particularly in Malaysia are greatly dependent on the water from rivers, the current status of AgNPs in the water system should be well-understood so that a probable water treatment can be carried out.

Scholarly literature has shown that AgNPs are present in numerous water sources with different concentrations. In general, a 5 to 95% of the total amount of AgNPs in the commercial products can be released into the sewage treatment plant^8^. Concentrations of AgNPs in the Connecticut undeveloped headwaters, the river from industrialized, and river from urban areas were found as 5, 25, and 100 ng L^−1^, respectively^13^. In the Texas river, the concentrations were in the range of 0.01 to 62 ng L^−1 ^^14^. On the other hand, there were 0.4 to 6.4 ng L^−1^ of AgNPs in the Trinity River estuary (Galveston Bay)^14^. Moreover, it was estimated that the concentration of AgNPs in the Rhine, a large European river, was in the range of 4 to 40 ng L^−1^ ^15^. The above-mentioned studies are undeniably significant for the monitoring of AgNPs content in the United States or Europe water environment. Noticeably lacking, however, are studies on the current status of AgNPs in Malaysia in terms of their fate and properties in the water bodies such as rivers and sewage treatment plants, which commonly receive the effluent from houses or industries directly.

Several methods such as aeration, coagulation, and adsorption have been implemented to remove AgNPs from the water. The aeration method is more complicated than the coagulation and adsorption since sequencing batch reactors are employed and the process is rather time-consuming. The coagulation method involves the use of toxic materials as coagulants such as aluminum sulfate, ferric chloride, poly aluminum chloride, and polyferric sulfate that impose potential threat as new pollutant sources in the water. The adsorption is seemed to be a safe procedure for removal of AgNPs from the water since it circumvents the complicated procedure while free of toxic materials use. Due to this positive attribute, the development of the synthetic, commercial, and natural materials as adsorbent has been intensively carried out. Nonetheless, there exists a heightened concern about the long-term adverse impact of synthetic materials because of their common association with toxicity. Although commercial materials are easily obtainable, their cost can be uneconomic when applied in large scales. Hence, a new material should be adopted to address the aforementioned shortcomings.

To date, materials in the previously under-explored activated carbon form are becoming more popular in the removal of pollutants that utilizes natural resources. Although numerous types of activated carbons have been proposed as an adsorbent for water treatment purposes, their use as the nanoparticle removal agent is lacking in the scientific literature and hence not well-understood. Nanoparticles are generally explored as the adsorbent but not adsorbate. It is more challenging to use nanoparticles as the adsorbate since they should be cleaned of ligand. Previous work found that the adsorption capacities of citrate-coated AgNPs on inorganic such as barium sulfate depend greatly on the ligand concentration^16^. It was well-established that the adsorption mechanism was firstly controlled by electrostatic interactions between nanoparticles and solid surfaces. However, the mechanism is probably different when the activated carbon, which is categorized as a hydrophobic adsorbent, is employed to remove AgNPs. This was observed when the Norit^®^ CA1 was employed as the commercial activated carbon^17^. Their investigation found that the steric repulsion by ligand shell failed to prevent the adsorption of AgNPs on the commercial activated carbon surface. This suggests that the electrostatic interaction force is not suitable to describe their interaction mechanism.

To extend the advantages of activated carbon as a promising AgNPs adsorbent, activated carbons derived from natural materials such as coconut and oil palm shells were investigated in this work. The objectives of the present work are: (1) to monitor the current situation of AgNPs in the water environment in Malaysia, (2) to propose a natural material for their removal, and (3) to investigate AgNPs adsorption mechanisms by evaluating 15 kinetics adsorption models. It is useful to note that there was an inconsistency in the scholarly literature exhibiting that the existing kinetic models did not perform well for a wide variety of experimental data. In addition, in some previous studies, only two to four kinetics models have been compared and assessed. Thus, it is challenging to find a general model to describe the kinetic behaviors of the adsorption of AgNPs. This study evaluated a series of commonly employed mathematical models. Outcomes from this study are beneficial in offering a candidate model that can be employed to characterize the kinetic behaviors of a wide variety of experimental AgNPs adsorption data in the future. In this work, wastes such as coconut and oil palm shells were used due to their abundant availability in Malaysia. Although other natural resources such as peat, coal, wood, sawdust, and bagasse can be used to produce activated carbon, the use of coconut and oil palm shells can be advantageous due to their high density, high purity, virtually dust-free nature. Also, carbons produced from their shells are harder and more resistant to attrition compared to grain or coal as raw materials^18^.

## Results

### Water physio-chemical indicators

Generally, drinking water can be derived from two basic sources such as surface water and groundwater. At present, surface water from the lake or river is the major source of drinking water in Malaysia compared to groundwater due to its availability and accessibility. To measure the water quality, physical and chemical indicators are commonly used. The relevant indicators of interest in this study are temperature (°C) and pressure (mmHg) (as physical indicators) as well as dissolved oxygen (mg L^−1^), conductivity (μs cm^−1^), pH, and nitrate (mg L^−1^) (as chemical indicators). Table 1 lists the aforementioned indicators for all water samples. There was no significant difference in terms of values for the temperature, pH, and pressure at all locations. In addition, the dissolved oxygen of the water samples from rivers was higher compared to samples from STPs.

**Table 1:** Physical and chemical indicators of all water samples.

Indicator	STP 1	STP 2	River 1	River 2
*T* (°C)	29.40 ± 0.00	29.60 ± 0.00	29.17 ± 0.06	28.37 ± 0.06
*P* (mmHg)	756.53 ± 0.06	756.60 ± 0.00	755.83 ± 0.06	757.00 ± 0.10
*DO* (mg L^−1^)	0.29 ± 0.07	0.24 ± 0.08	1.64 ± 0.16	1.48 ± 0.62
*C* (μs cm^−1^)	476.90 ± 15.38	574.27 ± 0.46	290.97 ± 0.50	305.47 ± 23.73
pH	7.29 ± 0.05	7.89 ± 0.01	7.06 ± 0.01	6.71 ± 0.12
Nitrate (mg L^−1^)	34.02 ± 23.65	41.15 ± 7.22	3.60 ± 0.26	22.99 ± 1.59

It is well-known that the increased level of dissolved oxygen can provide a higher possibility for aquatic life. The dissolved oxygen is highly beneficial for the survival of all aquatic organisms, not only for fish but also for invertebrates such as crabs, clams, and zooplankton. Moreover, it was observed that the conductivity and nitrate in the rivers for this study were lower than those from STPs. It was established that a high nitrate concentration can cause an excess algae growth in the rivers. Consequently, this condition can deplete oxygen concentration, resulting in the death of fish, aquatic organisms, and odor problems. The measured indicators have evidently shown that the water quality of the rivers was in good condition compared to STPs. Hence, it is clear that the water was treated before released into the rivers.

### Fate of AgNPs in the water environment

Since AgNPs are widely explored for various commercial products, their release into the environment is an issue of concern. AgNPs can be released into the water system through several ways such as from washing machine or from residual industrial materials. It is defined here that the functional zone surrounding STP 1 and river 1 is an industrial or commercial area and a residential area for STP 2 and river 2. The present study has found that the concentrations of AgNPs in STP 1, STP 2, river 1, and river 2 are in the ranges of 0.40 ± 0.44 to 15.38 ± 5.95, 0.13 ± 0.03 to 20.02 ± 0.56, 0.13 ± 0.06 to 10.16 ± 1.32, and 0.10 ± 0.00 to 9.63 ± 0.80, respectively, as listed in Table 2.

**Table 2:** Concentration of AgNPs obtained from ICP-OES assessment.

Location	Sampling event			
	1 (mg L^−1^)	2 (mg L^−1^)	3 (mg L^−1^)	4 (mg L^−1^)
STP 1	15.38 ± 5.95	0.47 ± 0.12	0.40 ± 0.44	0.53 ± 0.31
STP 2	20.02 ± 0.56	0.53 ± 0.31	0.33 ± 0.32	0.13 ± 0.03
River 1	10.16 ± 1.32	0.13 ± 0.06	0.23 ± 0.12	0.13 ± 0.06
River 2	9.63 ± 0.80	0.13 ± 0.06	0.30 ± 0.26	0.10 ± 0.00

It is obvious that there is a temporal variability of the concentration of AgNPs in the rivers and STPs. In general, the highest concentration is reached in monitoring event 1 (July) compared to others monitoring events (September, October, November for events 2, 3, and 4, respectively). Temporal variability in AgNPs concentrations can be principally explained by temporal variability in the water discharge. It was established that a higher water discharge from the sewage effluent commonly causes a higher AgNPs dilution^19^. A high AgNPs concentration in July is customarily due to a low water discharge. It is also important to note that Peninsular Malaysia has relatively dry weather (dry season) for that month due to the Southwest monsoon. In addition, relatively high temperatures are common in Malaysia in that particular month, which is primed for feeding and reproduction of many aquatic organisms^19^. These findings are in line with the previous work through simulation study that the highest concentrations of AgNPs in surface waters across Europe were found in July^19^.

### BET of activated carbon

The present work found that the total surface area, total pore volume, and average pore width for ACfOPS were 503.12 m^2^ g^−1^, 0.28 m^3^ g^−1^, and 0.55 nm, respectively. As a comparison, the total surface area, total pore volume, and average pore width were 538.17 m^2^ g^−1^, 0.22 m^3^ g^−1^, and 0.55 nm, respectively, for ACfCS. In general, their physical characteristics were not significantly different. Also, when compared with other studies, the presently proposed activated carbons are comparable^20^.

The pore sizes of the activated carbon can be categorized as micropores, mesopores, and macropores if the widths of the pores are <2 nm, 2–50 nm, and >50 nm, respectively^21^. Therefore, the prepared activated carbons in this study can be classified as materials having pores in the micropores category. In the adsorption mechanism, particularly that employing an activated carbon, its adsorption capacity principally depends on the number of micropores in the activated carbon. It is directly correlated to the micropore volume in the activated carbon. Relating to the current study, the presence of micropores in the activated carbon could enhance the adsorption of AgNPs that generally have sizes in the nanometer range via providing a wider surface for settlement. Therefore, the mechanism of AgNPs deposition on the activated carbon is not only controlled by the surface reaction but also their mobility into the micropores.

### SEM of activated carbon

**Figure 1:**
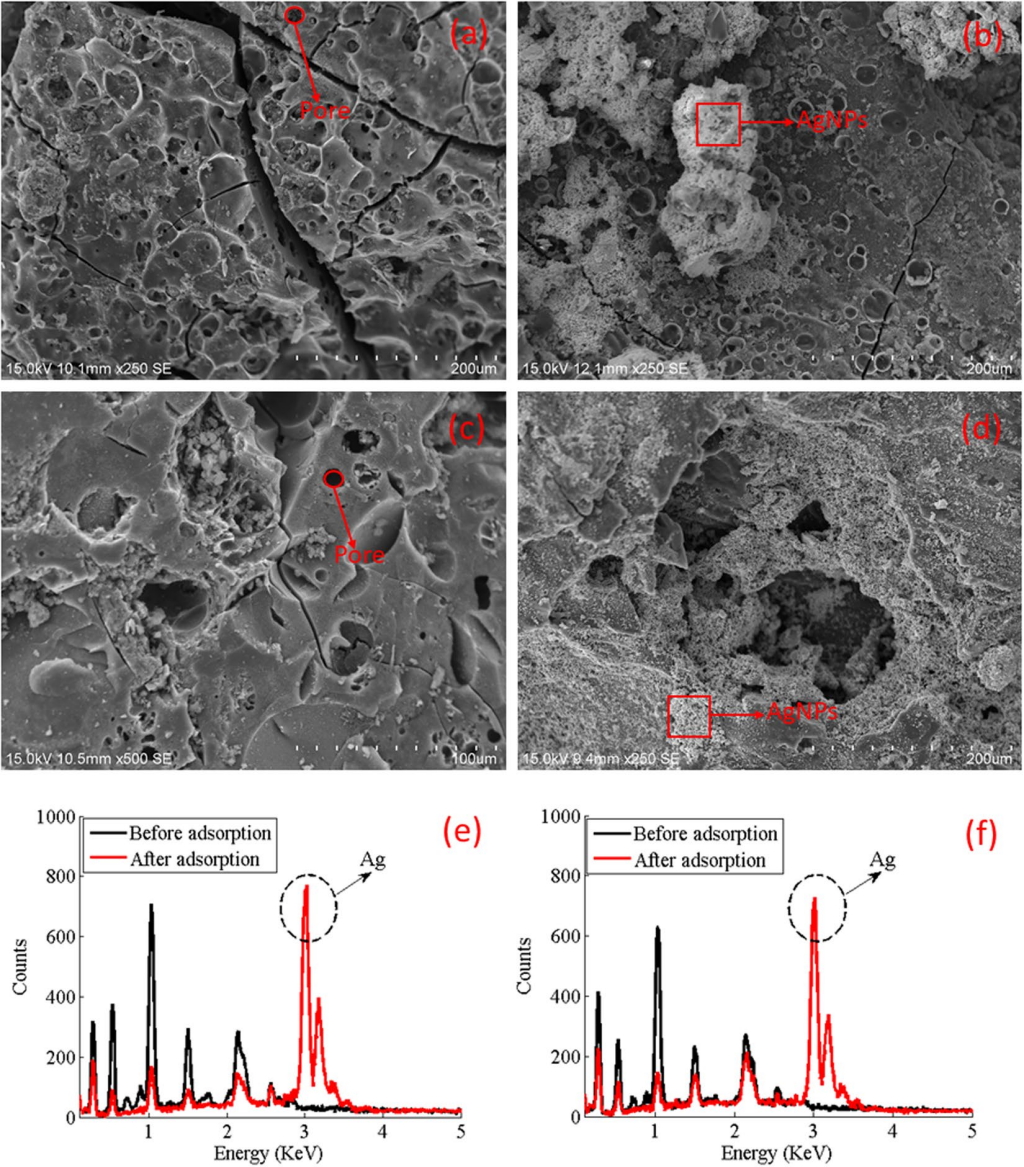
(**a**) SEM of ACfOPS before adsorption, (**b**) SEM of ACfOPS after adsorption, (**c**) SEM of ACfCS before adsorption, (**d**) SEM of ACfCS after adsorption, (**e**) EDX spectra of ACfOPS before and after adsorption, and (**f**) EDX spectra of ACfCS before and after adsorption.

Figure 1(a) shows the surface morphology of ACfOPS before adsorption of AgNPs. It is obvious from the figure that smooth areas are reflected by a series of oval lines. Various pores are also clearly identifiable on the surface of the activated carbon. In addition, it can be seen that a few cracks occur in Fig. 1(a). There are several noticeable pores on the activated carbon wall at the cracks. Figure 1(b) shows the close-up view of ACfOPS after adsorption of AgNPs. Based on the analysis of the images, two distinct types of surface can be detected. It is clearly identifiable on the surface of the activated carbon that after adsorption process the surface tends to be rougher than that before adsorption. It clearly indicates that AgNPs are attached to the surface of the presently fabricated activated carbon.

As a comparison, surface morphology characteristics of ACfCS before and after adsorption of AgNPs are presented in Fig. 1(c) and (d), respectively. Before adsorption, as shown in Fig. 1(c), there are two obviously distinctive regions, namely, smooth and rough areas. A similar smooth pattern can be observed for ACfOPS before adsorption of AgNPs, which is characterized by the existence of a series of oval lines. In addition, rough areas can be identified as a set of particle chips enveloping the surface of the activated carbon. A few pores also can be identified on the activated carbon surface. After adsorption, as shown in Fig. 1(d), a noticeable change in the activated carbon surface can be observed. Almost all activated carbon surface is enveloped by AgNPs. Therefore, its surface can be characterized as rougher compared to before adsorption.

### EDX of activated carbon

**Figure 2:**
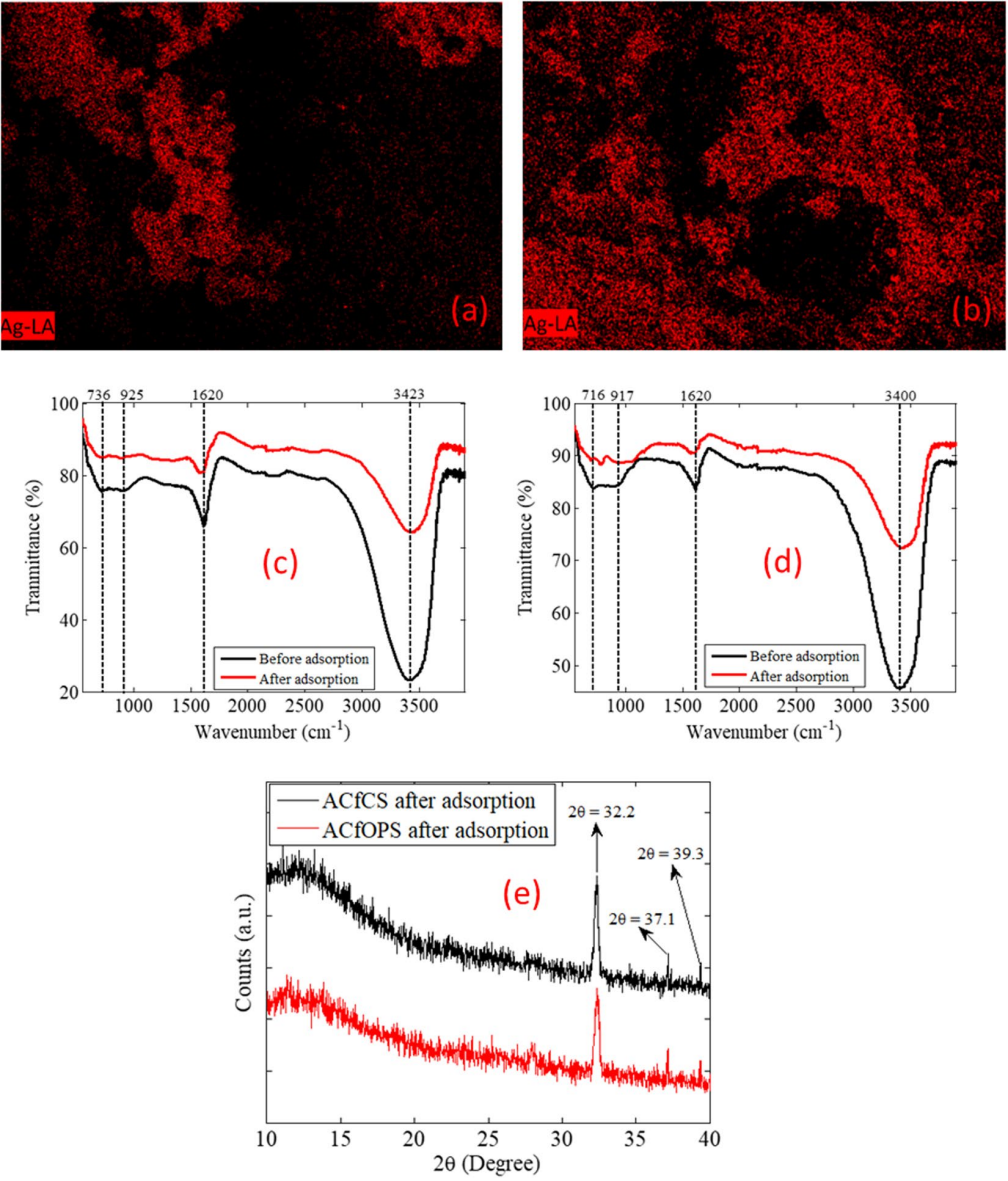
(**a**) AgNPs distribution on ACfOPS, (**b**) AgNPs distribution on ACfCS, (**c**) FTIR spectra of ACfOPS, (**d**) FTIR spectra of ACfCS, and (**e**) XRD spectra of ACfOPS and ACfCS.

EDX was employed to analyze elements of the produced activated carbon before and after adsorption of AgNPs. Figure 1(e) and (f) show the EDX spectra of ACfOPS and ACfCS, respectively. For ACfOPS sample as shown in Fig. 1(e), EDX spectra reveal a significant change in terms of count intensity before and after adsorption. Clearly, the silver element can be observed in the EDX spectra after the adsorption of AgNPs, indicating the presence of metallic AgNPs on the activated carbon surface. A similar pattern can also be observed in the EDX spectra of ACfCS as depicted in Fig. 1(f). The increase in count intensity indicating the presence of AgNPs is also observed in the EDX spectra after adsorption process. In general, a decreased count intensity for other elements is possibly due to the whole sample was almost covered by the metallic AgNPs causing a reduction in the interaction with the source of X-ray. It was well-established that the EDX analysis relies on an interaction of X-ray excitation with the sample. It has also been ratified that the count intensity of EDX spectra in the region of silver is high compared to other elements. For comprehensive verification, a mapping analysis was carried out as depicted in Fig. 2(a,b). It is apparent that the silver element dominates the surface of the samples.

### FTIR spectra of activated carbon

FTIR spectra were characterized to determine the surface groups on ACfOPS and ACfCS both before and after adsorption of AgNPs as depicted in Fig. 2(c,d). For ACfOPS, as shown in Fig. 2(c), it is clear from the figure that there are four obvious peaks at 3423, 1620, 925, and 736 cm^−1^. The peak at 3423 cm^−1^ can be correlated to the O–H stretching vibration in alcohols. A sharp peak at 1620 cm^−1^ is associated with the C=C stretching vibration in ketones. A weak peak at 925 cm^−1^ can be assigned to the P–O stretching vibration. The P–O stretching may come from the presence of phosphorus content in the raw material. A small peak at 736 cm^−1^ is related to the C–H out-of-plane bending in benzene derivatives. It was well-established that the main organic functional groups present in the palm oil shell were carbonyl groups (ketone and quinone), ethers, and phenols^22^. After adsorption of AgNPs, there were slight changes in terms of transmittance intensity and maximum peaks. For instance, the transmittance intensity of a peak of O–H stretching vibration in alcohols of the activated carbon was less intense after adsorption of AgNPs. Also, the maximum peak for that stretching vibration characteristic changed from 3423 to 3432 cm^−1^ after adsorption.

A similar characteristic is observed for the FTIR spectra of ACfCS. FTIR spectra of activated carbon derived from coconut shell can be observed in Fig. 2(d). In detail, peaks at 3400, 1620, 917, and 716 cm^−1^ are due to O–H, C=C, P–O, and C–H, respectively. These characteristics can be correlated to the stretching vibrations in alcohols, ketones, the presence of phosphorus content, and benzene derivatives, respectively. Agricultural residues, such as the presently proposed activated carbon, as derived from oil palm or coconut shells commonly compose almost the same functional groups such as phenol, aldehydes, alcohols, ethers, ketones, and carboxyl groups^22,23^.

### XRD of activated carbon

To understand the composition and structural change in the activated carbon after adsorption, the X-ray diffraction analysis was carried out. XRD spectra of ACfOPS and ACfCS are shown in Fig. 2(e). The maximum peaks of XRD spectra of ACfOPS at 2θ = 32.2°, 37.1°, and 39.3° are noticed. These spectra are identified as AgO characteristic with a monoclinic structure^24^. This lattice system has the cell edges having the relative lengths of *a* = 5.85, *b* = 3.48, and *c* = 5.49. Analogously, a similar pattern of XRD spectra for ACfCS can also be observed. Hence, this observation has confirmed that metal silver binds oxygen to form AgO.

## Discussion

Deposition mechanism of nanoparticles on activated carbon is a somewhat complex reaction. Several mechanisms have been proposed although all of them cannot exactly capture the complexity of the reaction^17,25^. In general, it was established that the deposition of nanoparticles on activated carbon greatly relies on the chemical surface of both activated carbon and nanoparticles, environmental condition, physical and mechanical parameters, and the preparation procedure. In this work, two activated carbons derived from different sources were proposed, namely, from oil palm and coconut shells. It is also possible that the activated carbons provide a slight change in the adsorption mechanism of AgNPs.

For ACfOPS, it can be found that AgNPs were deposited in several parts on the carbon surface, mostly as aggregates as shown in Fig. 1(b). Using this activated carbon, the present work proposes a series of possible rules of the deposition of AgNPs on its activated carbon surface. Firstly, the nanoparticles are instantly and completely deposited on the carbon surface. Secondly, AgNPs have potential to enter the activated carbon pores. Thirdly, AgNPs form clod by agglomeration phenomenon before attaching to activated carbon surface in the form as shown in Fig. 1(b). The distributions are also approved by the mapping of AgNPs in the activated carbon sample as depicted in Fig. 2(a). In comparison, the deposition mechanism of AgNPs on ACfCS is in a slightly different rule compared to ACfOPS. It has been observed that AgNPs were almost instantly and completely deposited on the carbon surface in mostly aggregates form, not as big clod as depicted in Fig. 1(d). Interestingly, almost all activated carbon surface was covered by AgNPs. This is confirmed by the mapping of AgNPs in the samples as shown in Fig. 2(b). Using this activated carbon, it is also possible that AgNPs can enter its pores and then settle.

Recent scientific literature has invoked somewhat contradictory findings on the interaction mechanism between nanoparticles as the adsorbate and natural materials as the adsorbent. On one hand, evidence of some scientific literature widely favors the case in which the adsorption process is dominantly initiated by electrostatic interactions between nanoparticles and solid surfaces^16,26^. On the other hand, the alternative study found that van der Waals forces or London dispersion forces are highly potential as the candidate to explain the adsorption mechanism of nanoparticles onto the activated carbon^17^. In the current work context, whether nanoparticles bind to the carbon, oxygen, or other constituents is still an inconclusively debated subject. Regardless of these proposed theories, it is clear that their interaction is widely controlled by the environmental conditions, nanoparticle charges, and molecules present in the activated carbon.

To understand the interaction mechanism for the current work, it is worthwhile to initiate discussion from the perspective of the nanoparticle transformation and their possible interaction with constituents of the activated carbon. It is well-known that AgNPs can be transformed in the water environment into a wide variety of elements such as Ag^+^ and Ag^2+^ depending on the physio-chemical conditions^27,28^. Their transformation is believed due to the reaction consumption of H^+^, which is usually favored at low pH^29^. By applying formula pH = −log[H^+^], the concentration of H^+^ can be measured as [H^+^] = 10^−pH^. It is certain that the concentration of H^+^ is higher at low pH or acid condition. For this reason, the pH changes of the prepared solution before and after adsorption were measured in the current study. It was found that the solution pH before adsorption was 3.29, which is defined as an acidic condition. Judging from this observation, the transformation of AgNPs from Ag° to Ag^+^ seems more favorable. After adsorption, the solution pH increased to 4.8 and 4.41 for the adsorption processes by ACfOPS and ACfCS, respectively, which are still in acidic condition. This mechanism was possible due to the surface protonation phenomenon. The activated carbon surface was more negatively charged during the adsorption process so that H^+^ ions presented in the solution configure layers in the surrounding of the activated carbon surface.

In terms of the interaction philosophy, it was well-established that H_2_O in the solution condition exists as H^+^ and OH^−^ in the following form:

1 $${{\rm{H}}}_{{\rm{2}}}{\rm{O}}\to {{\rm{H}}}^{+}+{{\rm{OH}}}^{-}$$

FTIR spectra shown in Fig. 2(c) and (d) have confirmed the presence of O-H molecule on the activated carbon. In solution, the molecule was dissolved as O^−^ and H^+^. Therefore, Ag^+^ attached to the activated carbon by binding with O^−^. This finding has also been confirmed by the XRD spectra exhibiting the presence of AgO after the adsorption process as readily shown in Fig. 2(e). Following this logic, it can be strongly implied that the electrostatic interactions between nanoparticles and O^−^ were the main forces that caused the adsorption of positively charged silver on the negatively charged solid surfaces of the activated carbon. By virtue of the aforementioned interaction philosophy, the chemical reaction of the adsorption of AgNPs on the currently proposed activated carbon can be expressed as:

2 $$[{{\rm{C}}}_{{\rm{n}}}{{\rm{H}}}_{{\rm{x}}+1}{{\rm{O}}}_{{\rm{y}}}]+{{\rm{Ag}}}^{0}\to [{{\rm{C}}}_{{\rm{n}}}{{\rm{H}}}_{{\rm{x}}}{{\rm{O}}}_{{\rm{y}}}]-{\rm{Ag}}+{{\rm{H}}}^{+}$$

**Figure 3:**
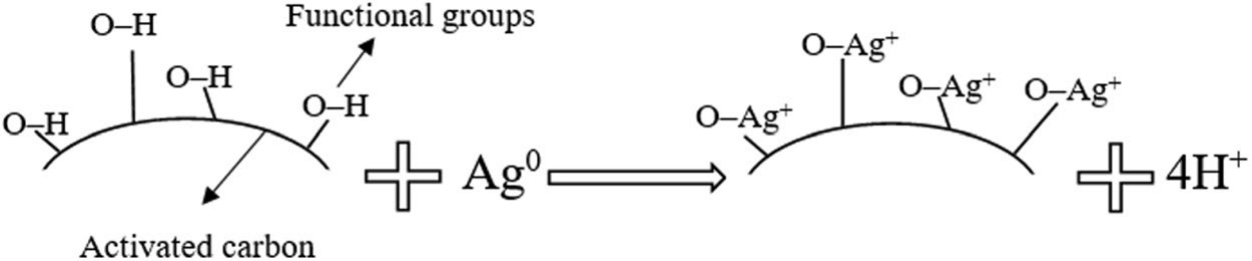
A proposed interaction mechanism between AgNPs and the currently proposed activated carbon.

For a complete overview, the interaction mechanism between AgNPs and the currently proposed activated carbon is proposed in Fig. 3.

One of the challenges in developing a mathematical model to represent a phenomenon of the adsorption of AgNPs is how the model can describe physically and chemically the event while making it simple for a widespread use. Although various models have been proposed, an apparent shortcoming is that the models are only applicable to a certain case examined by the proponents. Concerning with the aforementioned, the present work was dedicated also to evaluate a wide range of existing models to describe the adsorption of AgNPs via activated carbon materials. Table 3 presents all optimized parameters by employing ‘lsqcurvefit’ optimization of all presently evaluated models. It is apparent from the table that the prediction of *q*_*e*_ using the first-order, second-order, pseudo-first-order, pseudo-second-order, Avrami, mixed 1,2-order, exponential, Boyd, and fractal-like mixed 1,2-order models are close to the experimental data. However, alternative models such as fractal-like exponential, fractal-like pseudo-first-order, and fractal-like pseudo-second-order over-predict the experimental data. Although intraparticle diffusion, power, and Bangham models do not include *q*_*e*_, the models are also applicable for the present study.

**Table 3:** Optimized parameters of kinetic adsorption models for ACfOPS and ACfCS.

Model	Parameter	ACfOPS	ACfCS
First order	*q*_*e*_	0.199	0.190
	*k*_1_	0.243	0.180
Second order	*q*_*e*_	0.198	0.195
	*k*_2_	0.000	−0.001
Pseudo-first-order	*q*_*e*_	0.199	0.190
	*k*_1_	0.189	0.130
Pseudo-second order	*q*_*e*_	0.199	0.200
	*k*_2_	31.686	5.740
Intraparticle diffusion	*k*_*ip*_	0.000	0.001
	*c*_*ip*_	0.197	0.193
Power	*k*_*p*_	0.196	0.186
	*v*_*p*_	0.002	0.015
Avrami	*q*_*e*_	0.199	0.199
	*k*_*av*_	0.435	0.364
	*n*_*av*_	0.435	0.364
Bangham	*k*_*b*_	0.196	0.186
	*m*	349.208	65.000
Mixed 1,2-order	*q*_*e*_	0.199	0.200
	*k*	0.021	0.033
	*f*_2_	0.996	0.962
Exponential	*q*_*e*_	0.199	0.199
	*k*_*e*_	0.174	0.117
Fractal-like exponential	*q*_*e*_	0.422	0.327
	*k*_*fle*_	0.423	0.589
	*α*	0.005	0.028
Boyd	*q*_*e*_	0.199	0.199
	*B*	0.172	0.115
Fractal-like pseudo-first-order	*q*_*e*_	0.483	0.409
	*k*_*flfo*_	0.522	0.606
	*α*	0.003	0.021
Fractal-like pseudo-second-order	*q*_*e*_	0.565	0.661
	*k*_*flso*_	0.696	0.592
	*α*	0.004	0.021
Fractal-like mixed 1,2-order	*q*_*e*_	0.199	0.200
	*k*_*flfs*_	0.241	0.077
	*f*_2_	0.978	0.935
	*α*	0.592	0.848

**Figure 4:**
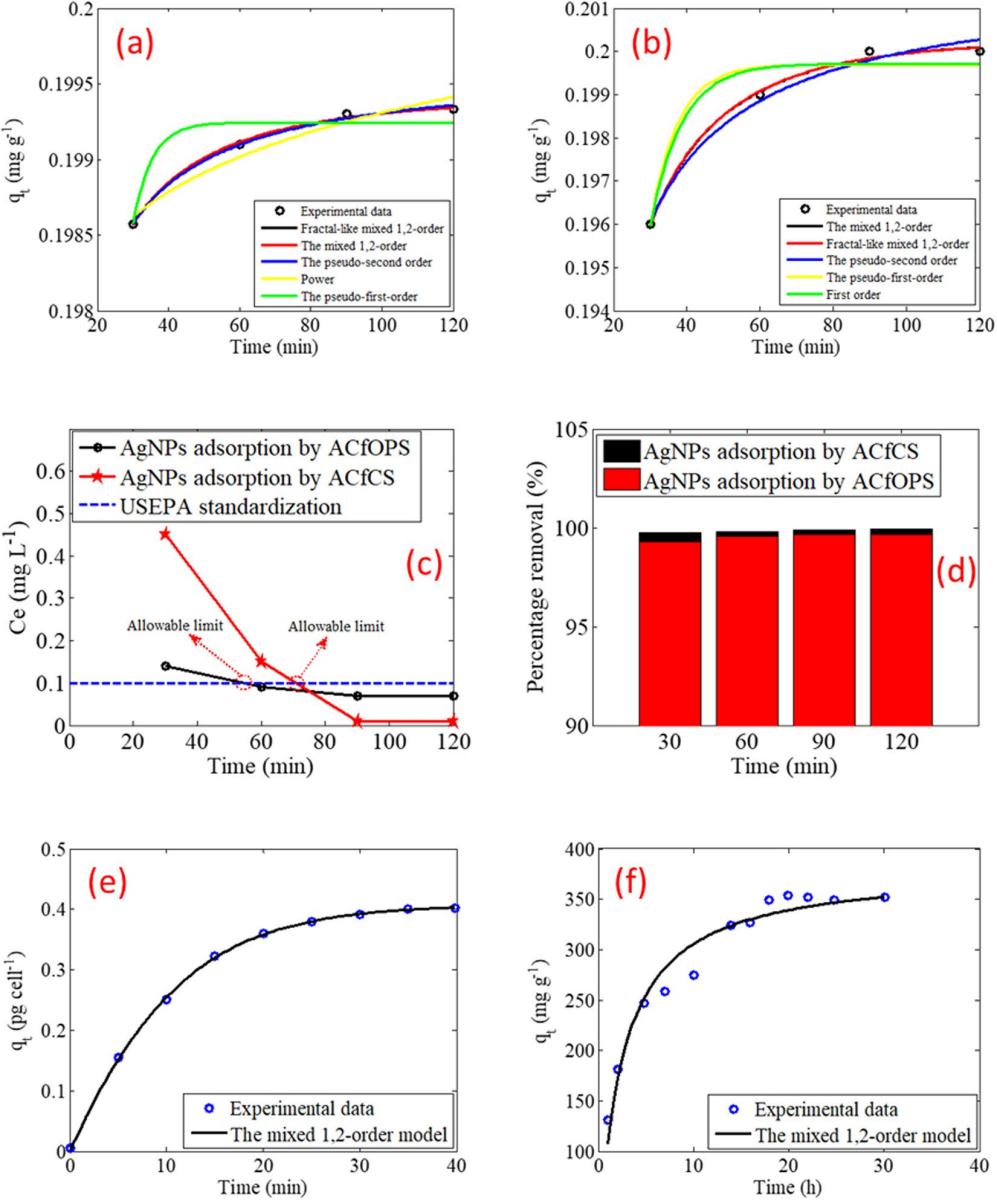
(**a**) The best five kinetic models for adsorption of AgNPs via ACfOPS, (**b**) the best five kinetic models for adsorption of AgNPs via ACfCS, (**c**) final concentration of AgNPs, (**d**) percentage of AgNPs removal, (**e**) performance of the mixed 1,2-order model for *Aeromonas punctata*, and (**f**) performance of the mixed 1,2-order model for Fe_3_O_4_@SiO_2_-PEI.

The study then assumed the following criteria: the model performance on the experimental data was considered very good if the average ranking < 3.75, good if 3.75 ≤ average ranking ≤ 7.5, satisfactory if 7.5< average ranking ≤ 11.25, and poor if 11.25 < average ranking ≤ 15. For AgNPs adsorption on ACfOPS as listed in Table 4, the mixed 1,2-order, fractal-like mixed 1,2-order, and pseudo-second-order models exhibit a very good performance. The Power and the pseudo-first-order models demonstrate a good performance. Those of Boyd, first-order, Bangham, exponential, fractal-like pseudo-first-order, Avrami, fractal-like exponential, and fractal-like pseudo-second-order display a satisfactory performance. However, the intraparticle diffusion and second order models show a poor performance. In detail, the best five performing models via ACfOPS outcomes are depicted in Fig. 4(a).

**Table 4:** Average ranking (AR) of all models for AgNPs adsorption on ACfOPS.

Model	*R*^2^ (Rank)	*RMSE* (Rank)	*E*_*max*_ (Rank)	*E*_*min*_ (Rank)	*MAPE* (Rank)	*MAD* (Rank)	AR
First-order	13	4	13	1	13	4	8.00
Second-order	15	15	15	15	15	15	15.00
Pseudo-first-order	11	5	11	2	11	5	7.50
Pseudo-second-order	3	1	3	8	3	1	3.17
Intraparticle diffusion	14	6	14	14	14	6	11.33
Power	7	7	7	12	4	7	7.33
Avrami	12	8	12	3	12	8	9.17
Bangham	8	9	8	13	5	9	8.67
Mixed 1,2-order	2	2	1	7	2	2	2.67
Exponential	10	10	9	4	9	10	8.67
Fractal-like exponential	9	11	10	5	10	11	9.33
Boyd	4	12	4	9	6	12	7.83
Fractal-like pseudo-first-order	5	13	5	10	7	13	8.83
Fractal-like pseudo-second-order	6	14	6	11	8	14	9.83
Fractal-like mixed 1,2-order	1	3	2	6	1	3	2.67

Analogous evaluation of the performance of all models to describe the experimental data of AgNPs adsorption via ACfCS is presented in Table 5. It has been found that there are only two models, namely, the mixed 1,2-order and fractal-like mixed 1,2-order that exhibit a very good performance. The pseudo-second-order, the pseudo-first-order, first-order, exponential, Boyd, and Avrami show a good achievement. Models such as fractal-like exponential, Bangham, and fractal-like pseudo-first-order reveal only a satisfactory performance. The Power, fractal-like pseudo-second-order, intraparticle diffusion, and second-order models are not suitable to describe the presently observed experimental data due to their poor performance. For a comprehensive overview, the best five performing models by employing ACfCS are depicted in Fig. 4(b).

**Table 5:** Average ranking (AR) of all models for AgNPs adsorption on ACfCS.

Model	*R*^2^ (Rank)	*RMSE* (Rank)	*E*_*max*_ (Rank)	*E*_*min*_ (Rank)	*MAPE* (Rank)	*MAD* (Rank)	AR
First-order	8	4	8	1	8	4	5.50
Second-order	15	15	15	15	15	15	15.00
Pseudo-first-order	6	5	6	4	6	5	5.33
Pseudo-second-order	3	3	3	8	3	3	3.83
Intraparticle diffusion	14	14	14	14	14	14	14.00
Power	12	9	12	13	13	9	11.33
Avrami	7	6	7	5	7	6	6.33
Bangham	13	10	13	9	10	10	10.83
Mixed 1,2-order	1	1	1	3	2	1	1.50
Exponential	5	7	5	6	5	7	5.83
Fractal-like exponential	9	11	9	10	9	11	9.83
Boyd	4	8	4	7	4	8	5.83
Fractal-like pseudo-first-order	10	12	10	11	11	12	11.00
Fractal-like pseudo-second-order	11	13	11	12	12	13	12.00
Fractal-like mixed 1,2-order	2	2	2	2	1	2	1.83

Since AgNPs were categorized as an emerging potential toxic contaminant, their removal from the water environment needs to be carried out. It was well-established that the United States Environmental Protection Agency (USEPA) has set the maximum of their contaminant level for drinking water standard at 0.1 mg L^−1^. As shown in Fig. 4(c), it is obvious that ACfOPS and ACfCS are capable to remove the concentration of AgNPs from the initial concentration of 20 mg L^−1^ down to the allowable limit (endpoint) by 54 min and 71 min adsorption processes, respectively. Although the final concentration achieved by ACfCS was higher compared to ACfOPS, for real application this study prefers to recommend the use of ACfOPS due to its faster performance in achieving the allowable limit concentration. It is highly beneficial for future industrial applications since the energy consumption, time consumption, and cost are expected to be greatly minimized. This good performance is possible due to the contribution of its pore volume that is slightly higher compared to ACfCS. Moreover, the present study has found that ACfOPS and ACfCS were able to remove AgNPs up to 99.6 and 99.9%, respectively, as depicted in Fig. 4(d).

The most surprising aspect of the present appraisal is that the mixed 1,2-order model consistently predicts a very good performance for the adsorption of AgNPs via both ACfOPS and ACfCS. It is hence ratified that the model is capable to offer an exact fit to our experimental data. Therefore, this model has the potential to be a prospective general model to describe the adsorption of AgNPs by numerous materials. Aligning to the aforementioned supposition, the present work then evaluated the performance of the model using the previous experimental data employing other natural and synthetic materials such as *Aeromonas punctata*^30^ and the poly(ethylenimine) functionalized core-shell magnetic mesoporous silica composites (Fe_3_O_4_@SiO_2_-PEI)^31^, respectively, the outcomes of which are as shown in Fig. 4(e,f). It is obvious that the model is also capable to demonstrate a very good performance for the examined data. In detail, *R*^*2*^, *RMSE*, *E*_*max*_, *E*_*min*_, *MAPE*, and *MAD* of the model are 1.00, 0.00, 0.14, 52.14, 6.33, and 0.00, respectively when *Aeromonas punctata* was employed as the adsorbent. For Fe_3_O_4_@SiO_2_-PEI, the corresponding values are 0.95, 14.97, 0.55, 18.37, 4.83, and 11.42, respectively.

In conclusion, the present work has comprehensively reported the current status of AgNPs in the water environment in Malaysia in terms of inspection, characterization, removal, modeling, and future perspective. This study has found that the concentration of AgNPs in the presently investigated sewage treatment plants was higher than that of the rivers with the highest concentration observed in July. In addition, FTIR, SEM, EDX, XRD, and BET analyses have been used to characterize the physio-chemical properties of the presently employed activated carbons for the removal of AgNPs. It has been found that ACfOPS was faster compared to ACfCS to achieve the allowable limit concentration permitted by the USEPA standardization. It was also ratified that the interaction mechanism between AgNPs and the activated carbon surface was mainly electrostatic forces interaction via binding of Ag^+^ with O^−^ presents on the activated carbon to form AgO, which was also confirmed by the XRD spectra data. The mixed 1,2-order model exhibited the best performance compared to other evaluated models. It can be concluded that the model offers an exact fit to our experimental data. Moreover, this model has the potential to be a general model candidate to describe the adsorption of AgNPs for numerous materials, its performance of which was very good when validated using other natural and synthetic materials. A further study should be carried out to assess the long-term performance of the proposed activated carbon before applying it on a large scale for future industrial purposes.

## Materials and Methods

### Materials

Oil palm and coconut shells as raw materials to produce activated carbon were collected from the palm oil plantations under the Federal Land Development Authority (FELDA) at Ulu Tebrau and Pasar Awam (a local market), Johor Darul Ta’zim, Malaysia, respectively. Silver nitrate (AgNO_3_) was purchased from QReC, Auckland, New Zealand. *Eleusin indica* was obtained from the surrounding area of Universiti Teknologi Malaysia, Johor Bahru, Malaysia. Zinc chloride (ZnCl_2_) was purchased from QReC, Auckland, New Zealand.

### Water sampling location

For inspection of the presence of AgNPs in the water environment, the water samples were collected from the local sewage treatment plants (STPs) managed by Indah Water Konsortium Sdn Bhd located at Desa Skudai (1.538247 N, 103.620674 E) and Taman Universiti (1.538346 N, 103.620681 E), Johor Darul Ta’zim, Malaysia. In addition, water samples were also collected from Melana River (1.535869 N, 103.623216 E) and Sekudai River (1.542408 N, 103.662191 E), Johor Darul Ta’zim, Malaysia. All water samples were then stored at a temperature of 4 °C for the next investigation. For brevity, Melana River, Sekudai River, STP at Taman Universiti, and STP at Desa Skudai are next abbreviated as river 1, river 2, STP 1, and STP 2, respectively.

### Preparation of silver nanoparticles solution

*Eleusin indica* extract was first prepared as follows. 18 g of fresh *Eleusin indica* was washed using tap water and then with ultrapure water three times each. The clean *Eleusin indica* was mixed with 200 mL ultrapure water in a 500 mL Erlenmeyer flask and the extraction process was conducted by boiling the mixture for 30 min before cooled to room temperature. The extracts were then filtered using a nylon membrane filter of 0.45 μm and stored in a fridge at a temperature of 7 °C for the next use. For AgNPs synthesis, a mixture of AgNO_3_ and ultrapure water with a concentration of 0.15 M in a 500 mL Erlenmeyer flask was prepared. 100 mL *Eleusin indica* extract was added to the 100 mL AgNO_3_ solution. The mixture was then stirred for 24 h at the rate of 100 rpm to complete the reduction of the silver ions to the nanoparticle.

### Preparation of activated carbon

For producing activated carbon derived from oil palm shell (ACfOPS), fresh oil palm shell was washed three times to remove any impurities using the tap water. It was then dried under the sun for 24 h to remove moisture content before ground and sieved to obtain pieces of irregular shapes with the sizes of <3 mm. To remove the excess moisture, the particles were then oven-dried at a temperature of 110 °C for 24 h. For the activated carbon preparation, the method proposed by Mahamad *et al*. was used as the basis^32^. Briefly, the dried shells were mixed with ZnCl_2_ at a weight ratio of 1:1 (dried coconut shell: ZnCl_2_) in a 500 ml beaker, in which the mixture was prepared using the ultrapure water. The mixture was left for 24 h at room temperature. It was then oven-dried at a temperature of 110 °C for 24 h. The carbonization process was conducted by heating the sample at a temperature of 500 °C for 1 h before cooled to room temperature. To remove any residual activating agent, the activated carbon was washed using the ultrapure water three times and finally oven-dried at a temperature of 110 °C for 24 h. A similar procedure was applied for activated carbon derived from the coconut shell (ACfCS).

### Characterization procedure

The concentration of AgNPs was analyzed using the inductively coupled plasma optical emission spectroscopy (ICPOES) (710 ICPOES No. my13270004) operated at a power of 1200 W with Argon flow rate of 15 L min^−1^. The adsorption-desorption isotherms of nitrogen were characterized using NOVAtouch 4LX from Quantachrome Instruments. The functional groups existed on the surface of activated carbon were analyzed using the Fourier transform infrared spectroscopy (FTIR) (PerkinElmer Frontier-GPOB model 96046) installed with the PerkinElmer Spectrum software by applying a spectrum wavelength in the range of 650 to 4000 cm^−1^ at a resolution of 4 cm^−1^ and accumulations of 10 scans at room temperature. Surface morphology of activated carbon was observed using the scanning electron microscopy (SEM) HITACHI S-3400N equipped with the Bruker Quantax software operated at a voltage of 15 kV. Elemental analysis was then performed using the energy-dispersive X-ray spectroscopy (EDX). X-ray diffraction (XRD) spectra were analyzed using the Bruker D8 Advance diffractometer with CuK as the radiation source, which has a wavelength of 1.54 Å.

### Adsorption experiments

The adsorptive capability of the produced activated carbons was evaluated by means of batch adsorption experiments using AgNPs. Activated carbons having particles with approximate sizes of 0.1 to 2 mm as obtained from the sieving procedure were employed in this investigation. AgNPs solution was diluted to obtain the maximum concentration similar to the actual maximum concentration of the water samples collected from rivers and STPs. In detail, a 10 mL AgNPs solution in a 50 mL plastic tube was prepared. Then, 1 g of the activated carbon was added to the solution. The adsorption process was investigated by magnetically stirring the mixture at a speed of 100 rpm for 30 to 120 min. The mixture was then filtered using filter paper. The supernatant was characterized to investigate the residue of AgNPs in the solution after adsorption. The particles retained on the filter paper was then used for the next characterization.

The residual concentration of AgNPs in the solution was calculated as a function of time in the range of 30–120 min, and the adsorption capacity was then calculated using the following formula:

3 $$q(t)=\frac{({C}_{i}-C(t))\times V}{W}$$

where *q*(*t*) is the adsorption capacity (mg g^−1^), *C*_*i*_ is the initial concentration (mg L^−1^), *C*(*t*) is the concentration at time *t* (mg L^−1^), *V* is the volume of AgNPs solution (L), and *W* is the mass of the activated carbon (g). In addition, the percentage of the removal was calculated using the following formula:

4 $${A}_{d}=(1-\frac{{C}_{f}}{{C}_{i}})\times 100 \% $$

where *A*_*d*_ is the percentage of removal (%), *C*_*i*_ is the initial concentration of AgNPs (mg L^−1^), and *C*_*f*_ is the final concentration of AgNPs (mg L^−1^).

### Theoretical kinetic models

In this work, fifteen kinetics models were used to evaluate the adsorption of AgNPs. These models are described as follows:

#### First-order model

The first-order model was initially proposed to describe the dynamic mechanism of lead and chromium removal on red mud from aqueous solution^33^. The model can be mathematically described as:

5 $${q}_{t}={q}_{e}-\exp (-{k}_{1}t)$$

where *q*_*t*_ is the adsorption capacity (mg g^−1^) at time *t* (min), *q*_*e*_ is the adsorption capacity at equilibrium (mg g^−1^), and *k*_1_ is the first-order rate constant (min^−1^). Therefore, the model has been continuously employed to describe other adsorption mechanisms^34^.

#### Second-order model

The second-order model was developed to describe the mechanism of the adsorption of cadmium ions onto bone char^35^. Mathematically, the model is given as^34,35^:

6 $${q}_{t}=\frac{{q}_{e}}{1+{q}_{e}{k}_{2}t}$$

where *k*_2_ is the second-order rate constant (min^−1^). The model was found reliable to accurately predict the equilibrium capacity of sorbent^35^. However, this model was found to deviate in terms of the rate of change of sorption from the data points after 6 h.

#### Pseudo-first-order model

The pseudo-first-order model was first proposed as a first-order rate model^36^. At present, it is well-known as the pseudo-first-order to differentiate the kinetic model based on the adsorption capacity from solution concentration^37^. The pseudo-first-order model can be presented using the following formula:

7 $${q}_{t}={q}_{e}[1-\exp (-{k}_{p1}t)]$$

where *q*_*e*_ is the adsorption capacity at equilibrium (mg g^−1^), and *k*_*p1*_ is the pseudo-first-order rate constant (min^−1^).

#### Pseudo-second-order model

Ho and McKay proposed a kinetic model to describe the adsorption of divalent metal ions onto peat^38^. In this model, the adsorption process of the second-order format is assumed. This model is commonly coined as the pseudo-second-order, which is mathematically expressed as follows:

8 $${q}_{t}=\frac{{k}_{p2}{{q}_{e}}^{2}t}{1+{q}_{e}{k}_{p2}t}$$

where *k*_*p*2_ is the pseudo-second-order rate constant (min^−1^).

#### Intraparticle diffusion model

Intraparticle diffusion describes the transportation of species from the bulk to the solid phase in a solution. It is commonly employed to explain the adsorption mechanism occurred in a porous material, which takes the following form:

9 $${q}_{t}={k}_{ip}\sqrt{t}+{c}_{ip}$$

where *k*_*ip*_ is the diffusion coefficient of the model (mg g^−1^ min^−1/2^) and *c*_*ip*_ is the intraparticle diffusion constant (mg g^−1^).

#### Power model

The nonlinear power model can be mathematically written as follows^34^:

10 $${q}_{t}={k}_{p}{t}^{{v}_{p}}$$

where *k*_*p*_ and *v*_*p*_ are the power constants of the model.

#### Avrami model

Avrami kinetic model has been widely used to describe the kinetic mechanism of several sorbates such as methylene blue or Hg(II)^39,40^. It is expressed as:

11 $${q}_{t}={q}_{e}[1-\exp {(-{k}_{av}t)}^{{n}_{av}}]$$

where *k*_*av*_ and *n*_*av*_ are the kinetic constants of the model (min^−1^) in the form of the fractional reaction order of the model (−).

#### Bangham model

Bangham kinetic model can be presented as follows^41^:

12 $${q}_{t}={k}_{b}{t}^{1/m}$$

where *k*_*b*_ is the constant of adsorption rate (mg g^−1^ min^−1^) and *1* */m* is the indicator of the adsorption intensity. This model can be employed to describe the adsorption of anionic and cationic dyes on activated carbon from aqueous solutions^41^.

#### Mixed 1,2-order model

Marczewski proposed a kinetic model derived from the pseudo-first-order and second order called the mixed 1,2-order model^42^, which is given as follows:

13 $${q}_{t}={q}_{e}\frac{1-\exp (-kt)}{1-{f}_{2}\exp (-kt)}$$

where *f*_2_ and *k* are the parameters of the mixed 1,2-order model. This model has been proven to successfully describe the adsorption of dye on mesoporous carbons compared to pseudo-first- or second-order model^42^.

#### Exponential model

An exponential form of the kinetic equation is also possible to describe the pattern of adsorption rate with time. The model can be expressed as:

14 $${q}_{t}={q}_{e}\,\mathrm{ln}[2.72-1.72\,\exp (-{k}_{e}t)]$$

where *k*_*e*_ is the constant of the exponential model.

#### Fractal-like exponential model

Haerifar and Azizian proposed a modification of the exponential model as^43^:

15 $${q}_{t}={q}_{e}\,\mathrm{ln}[2.72-1.72\,\exp (-{k}_{fle}{t}^{\alpha })]$$

where *k*_*fle*_ is the fractal-like exponential rate coefficient and *α* is the constant of the model. In this model, they hypothesized that this process occurred in systems whose solid surfaces were homogeneous. They found that the model can be applied for describing the kinetics of the adsorption for both homo- and heterogeneous systems.

#### Boyd model

Boyd model was used to predict the actual slowest step in the adsorption process^44^. The model is given as follows:

16 $${q}_{t}={q}_{e}[1-\frac{6}{{\pi }^{2}}\exp (-Bt)]$$

where *B* is the coefficient that covers the effective diffusion process and radius of the particles (min^−1^).

#### Fractal-like pseudo-first-order model

A modification of the pseudo-first-order model by introducing the fractal concept was also proposed^45^. The model was proposed as follows:

17 $${q}_{t}={q}_{e}[1-\exp (-{k}_{flfo}{t}^{\alpha })]$$

where *k*_*flfo*_ is the fractal-like pseudo-first-order coefficient and *α* is the constant of the model.

#### Fractal-like pseudo-second-order model

Haerifar and Azizian also proposed a modification of the pseudo-second-order model by introducing the fractal concept, which was mathematically described as^45^:

18 $${q}_{t}=\frac{{k}_{flso}{{q}_{e}}^{2}{t}^{\alpha }}{[1+{q}_{e}{k}_{flso}{t}^{\alpha }]}$$

where *k*_*flso*_ is the fractal-like pseudo-second-order coefficient and *α* is the constant of the model.

#### Fractal-like mixed 1,2-order model

Haerifar and Azizian also proposed a modification of the mixed 1,2-order model using the fractal concept^45^. The mathematical expression of the model was presented as:

19 $${q}_{t}={q}_{e}\frac{1-\exp (-{k}_{flfs}{t}^{\alpha })}{1-{f}_{2}\exp (-k{{t}_{flfs}}^{\alpha })}$$

where *k*_*flfs*_ is the fractal-like pseudo-second-order coefficient and *α* and *f*_2_ are the constants of the model.

#### Optimization procedure and statistical analysis

The MATLAB Optimization Toolbox curve fitting function, “lsqcurvefit”, was employed to optimize the model parameters. The Trust Region Reflective Newton algorithm was then utilized for the optimization problems^46^. Statistical indicators such as the coefficient of determination (*R*^*2*^), root mean squared error (RMSE), percentage of error in maximum estimated value (*E*_*max*_), percentage of error in minimum estimated value (*E*_*min*_), mean absolute percent error (MAPE), and mean absolute deviation (MAD) were employed to evaluate the performance of all models. The best model was chosen based on the minimum arithmetic mean rank. The mathematical formulae of all aforementioned statistical indicators are given as follows:

20 $${R}^{2}=1-\sqrt{\frac{\sum {({x}_{obs,i}-{x}_{model,i})}^{2}}{\sum {({x}_{obs,i}-{\bar{x}}_{obs})}^{2}}}$$

21 $$RMSE=\sqrt{\frac{{\sum }_{i=1}^{n}{({x}_{obs,i}-{x}_{{mod}el,i})}^{2}}{n}}$$

22 $${E}_{\max }=|\frac{{x}_{\max model}-{x}_{\max obs}}{{x}_{\max obs}}|\times 100 \% $$

23 $${E}_{\min }=|\frac{{x}_{\min model}-{x}_{\min obs}}{{x}_{\min obs}}|\times 100 \% $$

24 $$MAPE=(\frac{1}{n}\sum \frac{|{x}_{obs,i}-{x}_{model,i}|}{|{x}_{obs,i}|})\times 100 \% $$

25 $$MAD=\frac{1}{n}\sum |{x}_{obs,i}-{x}_{model,i}|$$

where *x*_*obs,i*_ is the observation data at time *i*, *x*_*model,i*_ is the prediction data at time *i*, and *n* is the number of data.
